# Proculturation shaped by social representations of academic migrants from Italy to the United States

**DOI:** 10.3389/fpsyg.2023.1173915

**Published:** 2023-05-04

**Authors:** Laura Dryjanska

**Affiliations:** ^1^Istituto Diplomatico Internazionale, Rome, Italy; ^2^Rosemead School of Psychology, Biola University, La Mirada, CA, United States

**Keywords:** Italian migrants, academic migrants, pre-migration expectations, proculturation, social representations, satisfaction with life, transnational families

## Abstract

**Introduction:**

Existing literature has highlighted the phenomenon of academic migrants leaving Italy for the United States with the hope of finding institutions that offer more opportunities for growth and recognition based on merit, as opposed to corruption, nepotism, and excessive bureaucracy. Likely, these may be the expectations of Italian academic migrants, who seem to be thriving and flourishing in their careers. This paper discusses proculturation of academic migrants from Italy to the United States, in the light of their expectations related to self-concept as well as social representations of North American university instructors from transnational families.

**Methods:**

In this study, 173 participants volunteered to provide information in an online survey that included their demographic profile, family situation, language ability, recalled pre-migration expectations and preparations, satisfaction with life, self-perceived stress, self-rated health, free responses to questions about major successes, challenges, and goals, as well as self-identification.

**Results:**

The results have shown that participants were indeed thriving in their careers and lives (majority scored high in satisfaction with life, health, realistic expectations and helpful per-migration preparations, while low in stress, also indicating work-related accomplishments and successes), but somewhat struggled with proculturation-related issues, frequently mentioned among major challenges.

## Introduction

Universities and colleges in the United States in general promote hiring migrants from various countries, as it increases diversity among faculty and instructors. Several studies have been conducted with graduate students who migrate from developing nations to industrialized countries (for example, Abdulai et al., 2021). There is also literature that concentrates on scholars who moved in the opposite sense, for example from Southern Europe to Mexico (Mendoza et al., 2020). Some researchers have explored the lived experiences of academic migrants—scholars born in other countries who work at universities and colleges at the destination country. The term “academic migrants” was introduced by Bönisch-Brednich (2014) who conducted a qualitative study with foreign-born instructors in New Zealand, which revealed their false assumption of similarity of universities across different countries.

According to recent reports (Licata, 2020), there are 283,350 registered Italian migrants in the United States (that constitute the seventh most frequent destination for Italians), with 5,291 new registrations in 2021. To date, no data is available with the number of academic migrants from Italy to the United States. While excelling at training researchers, academia in Southern Europe has been known for “challenging working conditions, low wages, low public investment in research, and a lack of fair competition for positions, all of which negatively affecting job prospects especially for those at early stages in their academic career” (Mendoza et al., 2020, p. 2). In general, Italy’s job market is associated with corruption, nepotism, and excessive bureaucracy (Varriale, 2021). It is not uncommon to refer to Italian researchers as “precarious” (Filippi et al., 2022), in a way constrained by the system to move abroad. Italian researchers’ top destination countries are the United States, the United Kingdom, and Australia, which paints a typical picture of brain-drain (Galan and Agasisti, 2014). While some argue for “brain circulation” among the academic community, several studies have demonstrated that many highly educated and specialized professionals do not “come back home,” but choose to live in a country different than their country of origin (Blachford and Zhang, 2014; Ortiga et al., 2018). This is especially the case of migrants employed by academic “centers” as defined by Altbach (2006, p. 124): “institutions with the funding, facilities, and qualified staff to pursue high quality research and teaching.” Such “centers” constitute “poles of gravity” within global spaces of networks of colleagues, frequently organized by profession, discipline, or technology (Mahroum, 2000).

This paper uses the theoretical foundation of the process of proculturation (Gamsakhurdia, 2018, 2019a,b, 2020) featuring the dialogical self-theory (Markova, 2000, 2003), as a key approach in the realm of social representations theory (Moscovici, 1961/1976; Moscovici, 1984; Moscovici and Markova, 1998; Moscovici, 2001), in order to explore the characteristics, wellbeing, achievements, challenges, and goals of academic migrants from Italy to the United States.

### Theoretical framework

Understanding wellbeing of academic migrants through the lenses of proculturation and social representations has so far been a relatively unexplored territory. More broadly, proculturation has been applied to understanding experiences of migrants and transnational families, especially in relation to their ongoing subjective construction of self (Gamsakhurdia, 2019a,^2021^), perceived loneliness and intergenerational belonging (Albert, 2021), negotiation of identity (Campill, 2021), and cultural identity (Ogoro et al., 2022), to mention just a few examples. Introduced by Gamsakhurdia (2018, p. 556), proculturation has been defined as “the process of reconstruction of the self after meeting and dialog with any kind of innovative cultural elements,” with the emphasis on the dynamic and continuous nature of this process. The integration of the dialogical nature of social representations (Moscovici and Markova, 1998) in the context of proculturation enables the acknowledgment of the involvement of mental systems in the interaction with the setting, while emphasizing the ability of the self to negotiate in the process of meaning making, while considering and recognizing the role of emotions (Gamsakhurdia, 2020). This conceptualization of proculturation in the light of novel social representations also recognizes the role of creativity in the process of the subjective construction of self and negotiation of meanings (Abdulai, 2020), in multiple roles played at work and in the transnational family settings. Albert and Barros (2021) note that the experience of migration places a person in multiple situations where they encounter new norms, practices, habits and traditions of receiving societies, which constitutes a context for developing new social representations that connect the inner and outer realities. In such situations, various characteristics of an individual may have an impact on proculturation. For example, language ability has been found to be an important factor of the adaptive proculturation process as migrants interact with others at their workplace (Boman, 2022), negotiating their identity on the continuum of able/unable to communicate in a foreign language. Correia and Watkins (2023) also highlight the role of language as a bridge in the process of semiotic adaptation that is a form of proculturation. Singh and Siddique (2021, p. 3) explain the relationship between language and proculturation pointing out that proculturation “is guided by the individual’s knowledge, experience and the expectation of the host culture. Thus, it is evident that the cultural change of migrants in the host culture is important both at the psychological and social levels. The acquisition of local language is one such vehicle that helps attaining them.” Likewise, among the characteristics relevant to proculturation, Gamsakhurdia (2019b) emphasizes the role of migrants’ expectations and knowledge when it comes to the depth of proculturation, especially in reaction to unfamiliar phenomena or situations. Such situations may occur at workplaces, but also when facing dynamics of being a part of a transnational family.

During the complex, dynamic, and ongoing process of proculturation, individual characteristics as well as the context interplay in a local and global scenario, for example including such worldwide phenomena as the aftermath of the COVID-19 pandemic (Correia and Watkins, 2023). A study of proculturation of migrant women in Israel highlighted the role of pandemic-related stress and protective factors related to identity negotiation (Dryjanska et al., 2021). Various roles that a person from a transnational family plays in life (for example identifying as a father and as a researcher) are dynamically relational, quite frequently involving a tension between societal and personal forces (Impedovo, 2020). Such tension can lead to stress of juggling multiple roles. An insight into these roles, or I-positions (Hermans, 2013), can provide more detail in terms of understanding the circumstances of proculturation. For example, a migrant mother with teenage children, who enters the United States as a researcher from Italy employed by a large private university, encounters multiple realities, including schools of her children, the workplace, public offices (where she needs to request a social security number or apply for a driver’s license), healthcare facilities, etc. Although similar settings exist in Italy, the interaction of I-positions may involve different dynamics as previously crystalized social representations become challenged (Grimell, 2018). For instance, the first visit to the doctor’s office in the United States involves a long time devoted to paperwork related to private health insurance that in turn depends on benefits at workplace. This connection between I-positions of a recipient of healthcare and an employee may not be as strong in Italy where the woman and her family were in the public healthcare system. Therefore, there is a new dynamic between the I-positions, where time becomes an important factor in how migrants negotiate, interpret, represent and experience their social realities (Gilodi et al., 2023). Throughout these experiences, each person may react differently in terms of emotions that impact the overall sense of wellbeing.

For academic migrants, job opportunities are frequently one of the main reasons why they decide to leave their countries of origin. In general, wellbeing at a workplace has been considered as one of the most crucial components of a person’s life, as work is very relevant to an individual’s self-identification (Apriyanti et al., 2021). This may become even more relevant to a migrant who makes important life choices seeking better opportunities in terms of their professional life. In positive psychology, wellbeing has often been differentiated between the two types; hedonic and eudaimonic, also considered in the context of a workplace (Straume and Vittersø, 2012). Hedonic wellbeing has to do with a person’s emotions when it comes to their life, which can be expressed as positive and negative affect, as well as life satisfaction (Diener, 1984). Eudaimonic wellbeing in terms of a workplace may have more to do with the intrinsic motivation or the actualization of one’s potential (Sanders et al., 2021); it focuses on optimal functioning and human growth (Bartels et al., 2019). Proculturation has been used as a framework where life satisfaction is an important indicator of migrants’ wellbeing (Afonso et al., 2022). Albert and Barros (2021) also consider life satisfaction in the light of transnational migrants’ identity when comparing proculturation of different generations. While migration has been considered by social scientists and mental health professionals among some of the most stressful life events that an individual can experience, there are limitations to an exclusive focus on negative mental health (Bak-Klimek et al., 2020). Overcoming the challenges related to proculturation in the context of a new workplace may increase life satisfaction and a sense of hedonic wellbeing, as well as intrinsic motivation to continue to excel and a sense of eudaimonic motivation. Furthermore, it has been shown that migration can have positive consequences for the individual wellbeing in later life, besides their economic living conditions (Gruber and Sand, 2022).

## Materials and methods

This article is based on quantitative research with 173 academic migrants from Italy to the United States. The study was approved on 7 February 2022 by the Institutional Review Board of Biola University, with an addendum to Protocol No. F17-004-JB. It is an extension of research conducted with Jewish participants who migrated to Israel from English-speaking countries (Zlotnick et al., 2020; Dryjanska et al., 2021) and participants who migrated to the United States from Spanish-speaking countries (Dryjanska and Zlotnick, 2021). The survey has been selected given the link between the proculturation framework of transnational families used in original studies (especially Dryjanska et al., 2021) that provided the theoretical foundation for the methodology applied in this sample. The adaptation to the target consisted of ensuring the appropriateness of questions by consulting with two members of the population of Italian academic migrants in the United States. The consultation resulted in the suggestion of adding three open-ended questions (described below) not used in original studies. The study was carried out in the late Spring and early Summer of 2022.

### Research instrument

The online questionnaire posted on Qualtrics was distributed among Italian academic migrants via mailing lists of various associations and organizations, such as the National Italian American Foundation, and through official diplomatic channels thanks to the assistance of the Italian Diplomatic Institute (*Istituto Diplomatico Italiano*). There was no monetary incentive to complete the survey; participants were encouraged to “make their voice heard” and informed that the study would advance knowledge about Italian academic migrants to the United States, their wellbeing, accomplishments, and challenges.

Questions included demographic information (e.g., year of birth, gender, education level, marital status, family situation, country of birth, reason (s) for migrating, date of migration, arrival location, professional status before and after migration); family network (e.g., how many and which family members accompanied the participant to the destination or have already lived in the destination country); questions about preparations to migrate and sources of information; a question to gauge the self-identification [“At this time in your life, what term (s) indicate how you self-identify (e.g., Italian, American, Italian-American)?” followed by a space for participant to provide some comments, reflections, or suggestions]; self-ranked state of health (using a five-point scale from “excellent” to “poor”); in addition to the four scales described in the following paragraphs. The study was administered in English. Fomenting the ecological validity of the research project, a member of the respondents’ community in California has provided consultation and feedback on each question and questionnaire as a whole, in order to propose a research tool that was considered appropriate, culturally relevant, non-offensive, safe, and readily understandable.

### Immigrants’ language ability

Immigrants’ Language Ability (ILA) consisted of three items and was rated on a five-level Likert scale, which resulted in scores ranging from 4 to 15. It has been used in the previous studies with English-speaking migrants to Israel, (Zlotnick et al., 2020) and Spanish-speaking migrants to the United States (Dryjanska and Zlotnick, 2021), in both cases with excellent internal consistency of Cronbach’s alpha = 0.95. Inspired with other scales that measure language acquisition, experience, and proficiency (Marian et al., 2007; Adamuti-Trache, 2013), ILA included the following questions: (1) “How would you rank your ability to speak English in everyday situations?”; (2) “How would you rank your ability to read English in everyday situations?”; and (3) “At work or when dealing with the U.S. bureaucracy, how would your rank your ability to speak English?” The scale rating ranged from “poor to non-existent,” “adequate,” “good,” “very good,” to “like a native-born.” In this study, internal consistency for the ILA scale was excellent (Cronbach’s alpha = 0.95).

### Realized expectations of acclimating to life after migration

Realized Expectations of Acclimating to Life After Migration (REALI) scale consists of three items and is aimed at measuring if the migrants’ initial recalled expectations were fulfilled following the migration experience. Participants consider how accurate was their knowledge about the social situation and ability to fit into the receiving society, as well as economic situation and ability to find work. There is also a direct question that invites participants to rate how realistic their expectations were regarding life in the United States. The total scores obtained in this study ranged from 4 to 15. The REALI was used in earlier research, and it attained good levels of internal consistency with Cronbach’s alpha = 0.77 (Zlotnick et al., 2020) and very good levels of internal consistency with Cronbach’s alpha = 0.83 (Dryjanska and Zlotnick, 2021). For this population, internal consistency for the REALI was very good (Cronbach’s alpha = 0.89).

### Perceived stress scale

Perceived Stress Scale (PSS-10) is a ten-item scale designed to measure perceived stress proposed by Cohen et al. (1983). Participants were asked to recall how often they thought or felt a certain way within the past month, rating the frequency on a five-point scale from 1 (“never”) to 5 (“very often”). Some examples of questions in PSS-10 include the following: “In the last month, how often have you been upset because of something that happened unexpectedly?,” “In the last month, how often have you felt nervous and stressed?,” “In the last month, how often have you felt that things were going your way?,” and “In the last month, how often have you felt that you were on top of things?” The total score ranged from 10 to 46. For this sample, internal consistency of the PSS-10 was very good (Cronbach’s alpha = 0.86).

### Satisfaction with life scale

Satisfaction with Life Scale (SWLS) measures global life satisfaction (e.g., “I am satisfied with my life,”) rather than a specific domain (Diener et al., 1985), using five items. Participants were asked to rate if they agreed with the following items on a seven-point Likert scale from 1 (“strongly disagree”) to 7 (“strongly agree”): “In most ways my life is close to my ideal,” “The conditions of my life are excellent,” “I am satisfied with my life,” “So far I have gotten the important things I want in life,” and “If I could live my life over, I would change almost nothing.” The total score ranged from 7 to 35. This scale has been used with participants from multiple ethnic backgrounds, obtaining excellent internal consistency (Cronbach’s alpha = 0.90) (Chang and Banks, 2007). SWLS had good internal consistency when used with some other samples (Lin et al., 2015), including migrants (Zlotnick et al., 2020; Dryjanska and Zlotnick, 2021). With this population, internal consistency of the SWLS was excellent (Cronbach’s alpha = 0.91).

The three open-ended questions concerned work-related challenges, successes, and ambitions: 1. What do you consider as your major professional or work-related challenge here in the United States?; 2. What do you consider as your major professional or work-related success here in the United States?; 3. What is your future goal or ambition that you are aiming for right now? Participants were provided space to include their response to these prompts, inviting brief statements related to each. They were free to mention one or more issues under each question, in their own words. The data was analyzed by two independent judges, creating categories inspired with the participants’ own words. For example, the category “language” was created based on many participants using the actual word “language” but also including “English” under this category. The inter-rater agreement was 94%.

## Results

**FIGURE 1:**
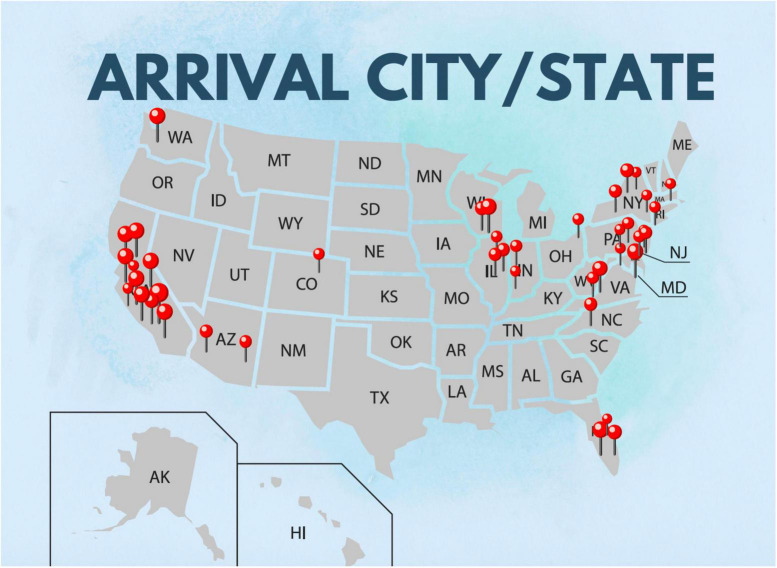
Arrival state of participants.

The majority of participants were born between 1951 and 1970, followed by 1981–1990, and could be described as seasoned or mid-career scholars. With a slightly larger proportion of men (55%), the sample consisted predominantly of married person (76.6%), followed by widowed, separated or divorced (10.8%), single (10.1%), and other (2.5%). The majority of respondents had adult children (on average two, followed by three and one child) and belonged to transnational families. The most common relocation decade was 2010s, followed by 2000s and 1990s, with the majority of participants coming to California and New York (see Figure 1). The majority of the respondents were either retired or active university professors, with 66.7% employed full-time and 18.3% employed part-time.

For the vast majority, their native language was Italian, with only several participants declaring English or another language as their native tongue. ILA results indicated that majority of participants felt confident in their ability to speak and read in English in diverse settings, describing it to be like a native-born American (64.9% in everyday situations when it comes to speaking, 72.4% in everyday situations when it comes to reading, and 59% when it comes to speaking at work). When prompted further, 32.1% of participants declared to speak English at home all the time, followed by mostly (27.2%), and sometimes (22.2%), with only 9.9% of participants replying with “rarely,” and 8.6% of participants replying with “never.” English was declared to be even more frequently used with friends than at home, with 35.9% of participants stating that they spoke English with friends all the time, followed by mostly (38.5%), and sometimes (23.1%), with only 1.3% of participants replying with “rarely,” and 1.3% of participants replying with “never.” Overall, the average score on ILA scale ranging from 1 to 15 was quite high: 13.26.

Expectations prior to migration are related to preparation and level of knowledge about the destination country. Academic migrants in general declared to had felt “prepared” (33.8%) or “very prepared” (25.7%) before moving to the United States; slightly over 40% of participants felt differently, with 23.0% replying “not very prepared” and 17.6%–“not prepared.” In a similar way, when rating their pre-move preparations at meeting their day-to-day life in the United States, 42.7% found them “helpful,” or even “very helpful” (24.0%); while 17.3% rated their preparations as “not very helpful” and 16.0%–as “not helpful at all.” When asked about the most helpful information sources, friends and family stood out as the common responses, followed by social networks and internet websites. Concerning pre-migration expectations, as measured with the REALI scale, the respondents felt that their expectations were indeed realistic (45.2%) or very realistic (17.8%), while for 20.5% the reply was “so-so,” followed by “not really” (13.7%) and “definitely not” (2.7%). Overall, the average score on REALI scale ranging from 1 to 15 was: 10.67.

In order to learn about participants’ health, the respondents rated their current health status on a five-point scale from “excellent” to “poor.” Overwhelming majority felt that their health was either “excellent” or “good,” with nobody rating it as “poor.” Self-perceived stress according to PSS-10 was on average 23.44, indicating typically experiencing fairly low levels of stress. Satisfaction with life according to SWLS was on average 26.81, with “I agree” standing out as the most common answer to questions such as “In most ways my life is close to my ideal,” “The conditions of my life are excellent,” “I am satisfied with my life,” “So far I have gotten the important things I want in life.” The cut-off benchmark for the scale is 26, which includes the two top categories of “satisfied” and “extremely satisfied,” out of a total of seven categories (Pavot and Diener, 1993).

Participants also had an opportunity to share about their work-related challenges, successes, and ambitions in a form of a free-text response. Overwhelmingly, in terms of challenges the American “culture,” as well as “mindset,” constituted the most common replies, followed by “language” and “communication,” as well as “visa” and “immigration” issues. The major professional success mentioned by the respondents had to do with obtaining a “PhD,” followed by “career” (often specified as “academic career,”) and “tenure.” When asked about their future goal or ambition, participants’ most common reply was “retirement” followed by “travel,” “saving money,” “coming back to Italy,” “writing a book,” achieving “stability,” carrying out “research projects,” becoming a “full professor,” “seeking new opportunities,” “winning an award,” moving “from ‘musts’ to ‘wants’,” and having “grandchildren’s affection.”

In terms of self-identification using a free-text response, 43.6% of participants described themselves as “Italian,” followed by 34.5% who chose the term “Italian-American,” 7.3% who opted for “Italian and American,” and the remaining percentage in other categories.

## Discussion

In this study, the proculturation framework (Gamsakhurdia, 2018, 2019a,^2020^) is applied to understand the wellbeing of workers in academic settings who migrated to the United States from Italy, taking into account their I-positions in various scenarios. Overall, the results revealed that participants had quite high levels of satisfaction with life (falling under the two top categories of “satisfied” and “extremely satisfied,” out of a total of seven categories), which is a component of the hedonic wellbeing (Diener, 1984). It is known from existing research with Italian academic migrants that they seek out institutions that offer more promising job prospects than those in Italy (Mendoza et al., 2020). North American universities are seen as hubs for academic centers (Altbach, 2006) and in spite of the fact that intra-European migration has become much less challenging and cumbersome over the last few decades (Cantwell, 2011), the United States appear as an attractive destination. Respondents in this study have in fact mostly lived in the destination country for at least over a decade, and at the time the survey was conducted they could be described as mature adults with well-developed careers. This may highlight the importance of time in the migrant trajectory of proculturation (Afonso et al., 2022). The participants’ self-rated health was predominantly “excellent” or “good” and they declared to have experienced low levels of stress, which are typically related to high levels of wellbeing and even thriving (Salimzadeh et al., 2017). It seems that in the light of linking the dimensions of the Perceived Stress Scale to the proculturation framework, the respondents in general succeeded in juggling multiple I-positions as academic migrants in transnational families, able to negotiate the meaning of who they are in a coherent yet complex self-concept. Furthermore, this study was carried out shortly after the COVID-19 pandemic noted in proculturation research as a major source of stress for migrants (Dryjanska et al., 2021; Correia and Watkins, 2023). Having dealt with the uncertainty of the unknown by remaining or even excelling in their position in academia in the United States might have brought a sense of satisfaction that stems from successfully facing challenges circumstances, which is also inherent to the proculturation dynamics. In other words, Italian academic migrants have been negotiating their identity in a particularly difficult scenario all over the globe. To link it with the dimensions of the PSS, because of facing the pandemic-related challenges as migrants, participants might have felt less upset because of something that happened unexpectedly during the last month, overall less nervous and stressed; on the contrary, more often feeling that things were going their way or that they were on top of things. Another possible indicator of successful proculturation may have to do with the professional successes, which reinforce I-positions that likely have a positive impact on self-esteem and overall wellbeing.

It is likely that participants were thriving in the academic career, as evidenced by how they identified the major professional success as obtaining a “PhD,” followed by “career” (often specified as “academic career,”) and “tenure.” The high satisfaction in life could have therefore be, at least in part, due to their positive experiences at workplaces. On the other hand, major challenges in life (American “culture,” as well as “mindset,” followed by “language” and “communication,” as well as “visa” and “immigration” issues) had to do with proculturation beyond academia in relation to multiple I-positions, and the migrants’ psychological and sociocultural adaptation (Cobb et al., 2019). The results pointed out that navigating American bureaucracy related to immigration has proved much more difficult to participants than the academic systems of promotion and tenure, as well as other career markers (such as “writing a book,” achieving “stability,” carrying out “research projects,” becoming a “full professor,”) mentioned in relation to future ambitions and goals, rather than challenges. Furthermore, “retirement,” “travel,” “saving money,” “coming back to Italy,” and “stability” might have been the goals or ambitions specific to the age group of mature adults preparing for the next stage in life, while strengthening the ties with the transnational families. It is interesting that “coming back to Italy” seems to become a more common option only after concluding the career in academia. In fact, during the recruitment process, several prospective participants justified their willingness to participate in relation to the disappointment with the work life reality in Italy, seen as an attractive destination for vacation and retirement. Their voices echoed the voices of those who, close to retirement or once retired, choose to move to Italy where they can enjoy the warmer climate, higher quality of life, and lower costs of living (King et al., 2019), coupled with the additional factors related to return migration in the post-pandemic reality (Genova and Zontini, 2020).

This research confirmed the existing findings that appropriate pre-migration preparations and accurate expectations contribute to flourishing during the migration experience (Dryjanska and Zlotnick, 2021). The majority of participants in this study declared to had felt “prepared” (33.8%) or “very prepared” (25.7%) before moving to the United States, rating their pre-move preparations at meeting their day-to-day life in the United States as “helpful” (42.7%) or even “very helpful” (24.0%). In a similar way, the respondents mostly felt that their expectations were realistic (45.2%) or very realistic (17.8%), which may mean that engaging in appropriate preparations and obtaining a realistic vision of academic career in the United States contributes to a better overall migration experience. It can also indicate that the majority of colleges and universities in North America are in fact accurately depicting the reality of work. The actual experiences at the workplace that are in line with the anticipated reality may involve less effort in terms of negotiation of meanings, as they can rely more on existing social representations, which in turn is probably less stressful (Sammut et al., 2012).

Finally, an attempt to reflect yet another aspect of proculturation consisted of asking each participant to choose a term to self-identify, with 43.6% of respondents describing themselves as “Italian,” followed by 34.5% who chose the term “Italian-American.” It seems that, while thriving in their academic careers, Italian migrants to the United States have mainly felt that their primary belonging matched the country of origin, which is also in line with the common response of hoping to return to Italy in the future, upon the completion of work life. This points into the usefulness of the framework of proculturation and emphasis on wellbeing when exploring the experiences of academic migrants, as this theoretical orientation can include reflections on flourishing and optimal functioning at work and in other settings (Krys et al., 2022). In other words, participants seemed to flourish and achieve high levels of wellbeing thanks to their workplaces in the United States, but for many of them the salient “Italian” component of identity would eventually be a drawing force to return to the country of origin, even if only to retire.

## Conclusion

Proculturation offers a useful theoretical framework for understanding wellbeing of academic migrants from Italy to the United States, taking into account multiple I-positions as professionals and components of transnational families, time, family configuration, and the emphasis on the dynamic process of meaning negotiation. It is noteworthy that overall high satisfaction with life, good health, and fairly low perceived stress seem to be pointing to the fact that participants were thriving in their careers away from home. It may seem that their expectations of fairness, opportunities for those who work hard, and promotion based on merit in North America, as opposed to nepotism, corruption, and inefficient bureaucracies associated with Italy (Varriale, 2021), were fulfilled during the migration experience. This research thus may be seen as a confirmation of other studies that highlighted the importance of realistic expectations prior to migration, conducted with Latinx in the United States (Dryjanska and Zlotnick, 2021) and English-speaking migrants in Israel (Zlotnick et al., 2020).

Majority of respondents consisted of mature adults with over a decade of living and working in the United States; unsurprisingly, their careers were frequently mentioned among major accomplishments, while immigration and proculturation-related issues tended to be considered as major challenges. Future ambitions had to do with retirement and stability or further steps in the academic career, such as promotion and tenure. Interestingly, the intention to return to Italy has been mentioned in the free-text responses to the question about their goals. Further research could include the question about return intentions as a part of the questionnaire and explore relationship with other variables, such as satisfaction with life, health, and self-perceived stress. Another interesting future direction consists of a longitudinal study, since this research project was carried out shortly after the COVID-19 pandemic that had likely influenced health considerations and travel plans. Assessing participants’ wellbeing over a period of time could shed additional light on understanding it in the context of their circumstances. Furthermore, from the perspective of positive organizational psychology, it could be helpful to have more information about presumably positive institutions, exploring which factors in American colleges and universities promote flourishing of academic migrants.

## Data availability statement

The raw data supporting by this conclusions of this article will be made available by the author, without undue reservation.

## Ethics statement

The studies involving human participants were reviewed and approved by the Institutional Review Board of Biola University. The patients/participants provided their written informed consent to participate in this study.

## Author contributions

The author confirms being the sole contributor of this work and has approved it for publication.
